# CXCR4 Inhibition Counteracts Immunosuppressive Properties of Metastatic NSCLC Stem Cells

**DOI:** 10.3389/fimmu.2020.02168

**Published:** 2020-10-02

**Authors:** Orazio Fortunato, Dimas Carolina Belisario, Mara Compagno, Francesca Giovinazzo, Cristiano Bracci, Ugo Pastorino, Alberto Horenstein, Fabio Malavasi, Riccardo Ferracini, Stefania Scala, Gabriella Sozzi, Luca Roz, Ilaria Roato, Giulia Bertolini

**Affiliations:** ^1^Tumor Genomics Unit, Fondazione Istituto di Ricovero e Cura a Carattere Scientifico (IRCCS) Nazionale dei Tumori, Milan, Italy; ^2^Center for Experimental Research and Medical Studies (CeRMS), Azienda Ospedaliera-Universitaria Città (AOU) della Salute e della Scienza di Torino, Turin, Italy; ^3^Laboratory of Immunogenetics, Department of Medical Sciences, University of Turin, Turin, Italy; ^4^Unit of Thoracic Surgery, Fondazione IRCCS Istituto Nazionale dei Tumori, Milan, Italy; ^5^Department of Surgical Sciences (DISC), Orthopaedic Clinic-IRCCS, A.O.U. San Martino, Genoa, Italy; ^6^Functional Genomics, Istituto Nazionale Tumori “Fondazione G. Pascale”, IRCCS, Naples, Italy

**Keywords:** metastasis initiating cells, non-small cell lung cancer, CXCR4, immunosuppression, CD73, adenosine, tumor associated macrophage (TAM), PD-L1

## Abstract

Cancer stem cells (CSCs) are functionally defined as the cell subset with greater potential to initiate and propagate tumors. Within the heterogeneous population of lung CSCs, we previously identified highly disseminating CD133+CXCR4+ cells able to initiate distant metastasis (metastasis initiating cells-MICs) and to resist conventional chemotherapy. The establishment of an immunosuppressive microenvironment by tumor cells is crucial to sustain and foster metastasis formation, and CSCs deeply interfere with immune responses against tumors. How lung MICs can elude and educate immune cells surveillance to efficiently complete the metastasis cascade is, however, currently unknown. We show here in primary tumors from non-small cell lung cancer (NSCLC) patients that MICs express higher levels of immunoregulatory molecules compared to tumor bulk, namely PD-L1 and CD73, an ectoenzyme that catalyzes the production of immunosuppressive adenosine, suggesting an enhanced ability of MICs to escape immune responses. To investigate *in vitro* the immunosuppressive ability of MICs, we derived lung spheroids from cultures of adherent lung cancer cell lines, showing enrichment in CD133+CXCR4+MICs, and increased expression of CD73 and CD38, an enzyme that also concurs in adenosine production. MICs-enriched spheroids release high levels of adenosine and express the immunosuppressive cytokine IL-10, undetectable in an adherent cell counterpart. To prevent dissemination of MICs, we tested peptide R, a novel CXCR4 inhibitor that effectively controls *in vitro* lung tumor cell migration/invasion. Notably, we observed a decreased expression of CD73, CD38, and IL-10 following CXCR4 inhibition. We also functionally proved that conditioned medium from MICs-enriched spheroids compared to adherent cells has an enhanced ability to suppress CD8+ T cell activity, increase Treg population, and induce the polarization of tumor-associated macrophages (TAMs), which participate in suppression of T cells. Treatment of spheroids with anti-CXCR4 rescued T cell cytotoxic activity and prevented TAM polarization, likely by causing the decrease of adenosine and IL-10 production. Overall, we provide evidence that the subset of lung MICs shows high potential to escape immune control and that inhibition of CXCR4 can impair both MICs dissemination and their immunosuppressive activity, therefore potentially providing a novel therapeutic target in combination therapies to improve efficacy of NSCLC treatment.

## Introduction

Lung cancer represents the first cause of cancer-related mortality worldwide (1). The predominant form of lung cancer is non-small cell lung cancer (NSCLC) for which available therapeutic options are largely ineffective because of its aggressiveness and diagnosis at metastatic phase (1). Treatment of NSCLC advanced stage disease used to rely on conventional platinum-based chemotherapy regimens that poorly impacted overall clinical outcome of patients, due to chemoresistance and frequent recurrence (2). Moreover, damage induced by chemotherapy in normal tissue has been proven to potentially cause the release of cytokines/chemokines that can sustain tumor cell survival and promote a receptive and immune-suppressive microenvironment able to chemoattract tumor cells at distant sites and foster metastasis initiation (3–5). Recently, immune checkpoint inhibitors (ICIs) have emerged as potentially revolutionary new drugs. First-line therapies combining cisplatin with ICIs may become the future mainstay of advanced NSCLC therapy (6–9). Unfortunately, a large number of patients still do not benefit from ICIs, and thus rationally designed combination strategies to extend ICIs effectiveness are mandatory (10).

We previously identified in NSCLC a subset of CD133+ lung cancer stem cells (CSCs), co-expressing CXCR4, endowed with stemness features and characterized by resistance to cisplatin and superior ability to seed distant site and initiate metastatic process (11–13).

CXCL12/CXCR4 axis has been described to play a pivotal role in CSCs maintenance, to guide tumor cell dissemination, and to foster chemoresistance (14–16). Cancer cells can up-regulate CXCR4 expression in response to extracellular adenosine, a potent immune suppressor molecule, thus acquiring increased ability to migrate and proliferate in response to CXCL12 (17, 18).

Due to its wide expression on several cell lineages, CXCR4 inhibition has been tested for different purposes and the CXCR4 inhibitor (Plerixafor) has been clinically approved for the mobilization of CD34+ hematopoietic stem cells for autologous transplantation in patients with lymphoma or multiple myeloma (19). Currently, several clinical studies are ongoing to test the efficacy of different CXCR4 inhibitors in metastatic patients with solid tumors (20–22). More recently, some studies have demonstrated that CXCR4 inhibition can reduce immunosuppression both by acting on Treg cells and myeloid derived suppressor cells (MDSC) that highly expressed CXCR4 receptor, overall resulting in the reactivation of T immune response against tumor cells (23–25). CXCL12/CXCR4 axis synergizes with CD38 to support migration as a central step in tumor disease progression (26). CD38 is a pleiotropic glycoprotein receptor with enzyme activity involved in the catabolism of extracellular nucleotides (27). Therefore, multifunctional protein CD38 can contribute to immune suppressor of T cell, activating the non-canonical adenosinergic pathway that provides AMP substrate to CD73 (28, 29).

CD73 can be expressed on cancer cells and different immune cell populations. This molecule dephosphorylates extracellular adenosine monophosphate (AMP) generating free adenosine, which contributes to the immune-suppressive and pro-angiogenic microenvironment at the tumor site (30, 31). It is known that adenosine is involved in tumor immune escape, and thus the block of CD73 enzymatic activity can reactivate an antitumor immune response (32) by synergizing with chemotherapeutic drugs known to promote immunogenic responses and enhance the therapeutic activity of ICIs (33–35). Anti-CD73 antibody has been demonstrated effective in reducing tumor growth and metastatization in mice (32, 35, 36). Remarkably, CD73 expression has been described as a poor prognostic factor for overall survival in NSCLC (37). A significant population of CD39+CD73+ myeloid derived suppressor cells, capable of inhibiting T and NK cell activity, has been shown in peripheral blood and tumor tissues of NSCLC patients (38).

Immunotherapy based on ICIs have achieved significant results in clinical practice, improving survival of patients with cancer (39). However, only a fraction of patients have shown long-term benefit, and the high rate of resistance still limits their efficacy (40). The mechanisms of resistance to ICIs are quite different, and among them the up-regulation of CD38 by tumor cells determines a functional impairment of CD8 T cells, with a consequent tumor immune escape (41). Chen et al. demonstrated that the co-inhibition of immune checkpoints and adenosine release improves anti-tumor immune response (41).

Interestingly, also CXCR4 inhibition results effective in reverting tolerogenic polarization of tumor microenvironment (42) and in restoring sensitivity to CTLA-4 and PD-1 checkpoints inhibitors (24, 43).

Here, we report that NSCLC CD133+CXCR4+ metastasis-initiating cells (MICs) are endowed with immunosuppressive properties allowing them to escape immune control, by the expression of high levels of PD-L1 and CD73/CD38 ectoenzymes, that mediate extracellular adenosine generation (28). We prove the ability of a new class of CXCR4 antagonists (44) to counteract the immune suppressive behavior of metastatic NSCLC stem cells, pointing at CXCR4 as novel target to prevent metastatic dissemination and immune escape mechanisms exploited by MICs.

## Materials and Methods

### Cell Cultures and Pharmacological Treatments

NSCLC cell lines (A549, H1299, H3122, SW900) were purchased from ATCC and cultured in adhesion in conventional medium, RPMI-1640 supplemented with 10% heat-inactivated bovine serum (RPMI 10%) (FBS, all from Lonza). Bronchial‐epithelial cells (HBEC3KT), immortalized by hTERT and CDK4, were obtained from Prof J. Minna (UT Southwestern) and cultured in Keratinocyte SFM (ThermoFisher).

To obtain sphere cultures, cells were plated in Ultra-Low Attachment plates (Corning) at a density of 10^4^ cells/ml in serum-free medium DMEM/F12 (Lonza), supplemented with commercial hormone mix, B27 (Gibco), EGF 20 ng/ml, bFGF10 ng/ml (PeproTech), and heparin 2 µg/ml, named Stem Cells Medium (SCM). Floating sphere cultures were expanded for 15 days in the above medium. Once a week, they were gently dissociated with Accumax (Sigma-Aldrich) and re-plated as single cells in fresh medium.

Adherent cells and dissociated spheroids were incubated with peptide R 1 µM for 2 h at 37°C at a density of 2.5x10^5^ cells/ml in respective complete medium. Next, the medium was removed and fresh medium was added and collected after 24 h to obtained cancer cell conditioned medium (CM).

PBMCs from healthy volunteers were plated at 1x10^6^ cells/well in well plates and incubated at 37°C for 4 h. After the incubation, non adherent cells (T cells) were removed and used for the experiments. At the same time, adherent cells (monocytes) were differentiated to macrophages for 7 days with 50 ng/mL of human M-CSF.

Stimulation of T cells was performed by Dynabeads Human T-Activator CD3/CD28 and cultured in 50% of CM from different cancer cell lines. According to cell lines, negative control of the experiment were T cells cultured in RPMI 10% or 50% Stem Cell Medium (SCM).

Spheroids were treated with different concentration of mAb anti-CD73 (10, 20, 50 ug/ml, clone CB73, generated and purified in house through a two-steps HPLC chromatography by FM) or Adenosine 5’-(α,β-methylene)diphosphate (APCP, at 25, 50, 100 uM, Sigma) every 48 h for 7 days.

### Flow Cytometry Analysis

To analyze tumor cell surface markers, single cell solution was washed in staining buffer (PBS1×+ 0.5% BSA+ 2mM EDTA) and incubated for 30 min at 4C° with the following antibodies: anti-human PE-CD133/1 (clone AC133/1Miltenyi Biotech), APC anti-human CXCR4 (44717 clone-R&D system), BB515 Anti-human CD73 (clone AD2), BB700 Mouse Anti-human CD38 (clone HIT2), BV421 Mouse Anti-human CD274 (PD-L1 clone MIH1), AlexaFluor488 Anti-Human HLA-ABC (clone DX17), and BV510 CD39 (clone A1) (all from BD Biosciences).

Primary tumor cell suspensions were obtained by digesting primary tumors, from consenting patients, with human Tumor Dissociation Kit (Miltenyi), subsequent filtering of dissociated tumor tissue on 100 μm pore cell strainer (Falcon), and erythrocytes removal by Red Blood Cell Lysis Solution (Miltenyi Biotech). Tumor cells were then stained with CD133, CXCR4, CD73, or PD-L1 (as specified above). Stromal cells were identified by staining for PE-Cy7 anti-human CD45, CD31, CD34 (eBioscience) and excluded by a negative gating strategy to perform tumor cell analysis.

To analyze the different subtypes of macrophages, cultured cells were washed in staining buffer and incubated for 30 min at 4°C with the following antibodies: Alexa488 anti-human CD206 (clone 15-2) (Biolegend) and PE anti-human CD163 (clone GHI/61) (Biolegend), APC anti-human CD14 (clone M5E2) (BD Biosciences).

For staining of T cytotoxic cells, lymphocytes were incubated in staining buffer with BV510 Anti-Human CD3 (HIT3a) and BB515 Anti-Human CD8 (clone Leu3a) for 30 min at 4°C; then the cells were fixed and permeabilized with BD Cytofix/Cytoperm™ Solution for 30 min at 4°C, washed in BD Perm/Wash buffer, and incubated with APC anti-human IFNγ (clone B27) (all from BD Bioscience), for 30 min at 4°C.

For analysis of Treg phenotype, T cells were first incubated with surface antibodies in staining buffer for 30 min at 4°C: BV510 Mouse Anti-Human CD3 (HIT3a), PE-Cy7 Anti-Human CD4 (clone Leu3a), APC Anti-Human CD25 (clone M-A251); then fixed and permeabilized with Transcription Factor Buffer Set, according to the datasheet instructions, and finally incubated with PE anti-Human FoxP3 (clone259D/C7) (all from BD Biosciences) for 30 min at 4°C. Tregs were identified within live cell gate as CD3+CD4+Foxp3+CD25^high^.

For all analyses, dead cells were excluded by the use of Fixable Viability Stain 780 (BD Horizon). Data were acquired with a FACSCanto cytometer (BD) and analyzed by FlowJo software V10.

### PBMCs Proliferation Assay

Two different tests were performed to assess T-cell proliferation: MTT and CSFE staining.

MTT assay: PBMCs derived from buffy coats were plated in a 96 well plate at 2x10^5^ cells/well in RPMI, 10% FBS. To induce proliferation PBMCs were stimulated with OKT-3 (7.5 μg/ml) and anti-CD28 (7.5 μg/ml) and cultured with 50% of CM from cancer cells for 72 h.

MTT assay was performed according to the manufacturer’s instructions (Sigma-Aldrich).

CSFE staining: T cells were incubated with CFSE (BD Biosciences) to a final concentration of 1.5 uM, for 8 min at room temp. The reaction was blocked by incubating cells in FBS. Stained T cell were plated at 1x10^5^ cells/well in 24 well plates with RPMI+10% FBS and stimulated with antiCD3/CD8 microbeads and CM from tumor cells (ratio 1:1). Unstimulated T cells, plated in RPMI 10% or SCM +RPMI 10% (ratio 1:1) according to different tested CM, represent the negative control of the experiments. After 72 h T cells were analyzed by FACS to assess the % of CSFE stained cells, which was inversely correlated to the proliferation rate.

### Migration/Invasion Assay

For migration assays, 50.000 cells/well were incubated with peptide R inhibitor of CXCR4 (1μM) or AMD3100 (10 μM) and seeded in 200 µl of RPMI-1640 medium supplemented with 1% FBS onto 8 μm-pore Transwell^®^ cell culture inserts (BD Falcon) in 24 well plate. The lower chamber was filled with 500 µl of RPMI supplemented with SDF-1 (50 ng/ml) as chemoattractant factor. For the invasion assay 1x10^5^ cells were plated onto 8 μm-pore Transwell^®^ cell culture inserts covered with 20μl of Matrigel, which was allowed to solidify at 37°C.

After 48 h (migration assay) or 72 h (invasion assay), cells on the top of the insert membranes were removed by gentle scraping with a sterile cotton swab while migrated/invaded cells in the lower side of the insert were fixed in methanol and mounted on slides using the VECTASHIELD Mounting Medium, containing DAPI. For each insert, cells in 4 random fields were counted by fluorescence microscope visualization at 20X magnification, and the values were averaged. Each experiment was performed in triplicate.

### Adenosine Quantification

Twenty-four hours before the adenosine assay, adherent cells were seeded on 24 well plates at a concentration of 5 x 10^5^/500 µL, while lung spheroid cells were transferred into 24 well plates in new medium, after have being cultured for 15 days (as previously described).

Culture medium was removed from adherent cells simply by pipetting, while spheroids cells were collected in Eppendorf tubes, centrifuged at low speed to pellet them down, and medium was removed. The cells and derived lung cancer spheroids were incubated with 100 µL STOP solution (EHNA 100 µmol/L, DYP 10 µmol/L, and 10 µmol/LDEF) (Sigma-Aldrich) for 15 min at 37°C and then treated with 100 µL AMP 100 µmol/L for 10 min at 37°C on a basculant. After incubation, the cells were collected in a tube containing acetonitrile (ACN; 1:2; 4°C), centrifuged (13000 *g* for 5 min at 4°C). Tubes were transferred into a Speed Vac (Eppendorf), to remove the supernatant, reconstituted in HPLC-grade water, and assayed or stocked at -80°C.

Chromatography analyses of the supernatant were performed with an HPLC (Beckman Coulter) fitted with a reverse-phase column (Synergi 4U Polar-RP80A; 150 x 4.6 mm; Phenomenex). Nucleotides and nucleosides were separated using a mobile-phase buffer (0.025 mol/L K_2_HPO_4_, 0.01 mol/L sodium citrate, 0.01 mol/L citric acid, adjusted with phosphoric acid to a pH of 5.1 and 8% acetonitrile (ACN) for 13 min at a flow rate of 0.6 mL/min. Ultraviolet (UV) absorption was measured at 254 nm. Chromatography-grade standards used to calibrate the signals were dissolved in PBS 1X, pH 7.4 (Sigma-Aldrich), 0.2 µm-filtered, and injected in a volume of 15 µL. The retention times (R_t_, in min) of standards were: AMP, 5.8; inosine (INO), 6.4; and adenosine (ADO), 10; using a R_t_ window of ± 5%. Peak area was calculated using Gold software (Beckman Coulter). Quantitative measurements were inferred by comparing percentage area of each nucleotide and nucleoside analyzed, as previously described (29).

### Real-Time PCR

Automating RNA isolation was a performed by Maxwell RSC using simplyRNA Cells Kit (Promega). Expression levels of IL-10 and CD73 genes were determined by Real-Time PCR, using TaqMan^®^ assays (Thermo Fisher) and normalized using the 2−ΔΔCt method relative to B2M, and results are expressed as mean ± SD. For each PCR reaction, 5ng cDNA input was added.

### Protein Extraction and Western Blot Analysis

Whole cell extracts were obtained from cell lines treated with 1 μM CXCR4 inhibitor using GST-FISH buffer (10 mM MgCl_2_, 150 mM NaCl, 1% NP-40, 2% Glycerol, 1 mM EDTA, 25 mM HEPES pH 7.5) supplemented with protease inhibitors (Roche), 1 mM phenylmethanesulfonylfluoride (PMSF), 10 mM NaF, and 1 mM Na_3_VO_4_. Extracts were cleared by centrifugation at 12,000 RPM for 15 min. The supernatants were collected and assayed for protein concentration using the Bio-Rad protein assay method. Twenty μg of proteins were loaded on 12% Mini-PROTEIN TGX gels (BIO-RAD), transferred on nitrocellulose membrane (GE Healthcare), and blocked with 5% skim milk (BIO-RAD). Primary antibodies for immunoblotting included monoclonal anti-rabbit NT5E/CD73 (D7F9A clone, Cell Signaling Technology, CAT NO #13160) and rabbit polyclonal anti-βactin (Sigma, CAT NO #A2066). Membranes were developed with ECL solution (GE Healthcare).

### Statistical Analyses

Statistical analyses were performed using GraphPad Prism version 6.0. Statistically significant difference between two groups was assessed by two-sided Student’s t-test. Statistical analyses among more than two groups was performed by one-way Anova with Tukey’s *post hoc* test. Data are expressed as means and standard deviation, unless otherwise indicated. Statistical significance was defined as a P value less than 0.05.

## Results

### Lung Cancer Metastasis Initiating Cells Highly Express PD-L1 and CD73 Markers

We initially investigated by flow cytometry the expression of PD-L1 and CD73 on surgically resected primary NSCLC samples (n=22), within tumor bulk population and CD133+ CSC subsets.

**Figure 1:**
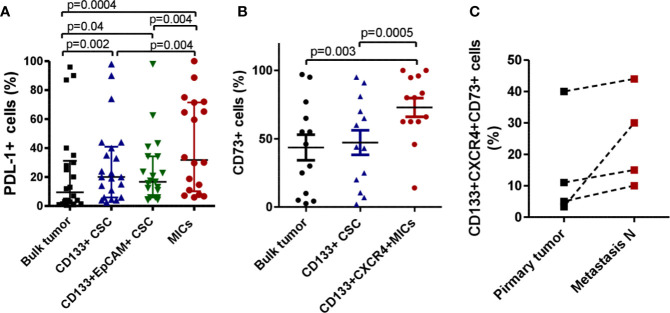
MICs highly expressed immunoregulatory markers. **(A)** FACS analysis of N=22 NSCLC primary tumors. PD-L1 expression was assessed within bulk tumor population and different subsets of CD133+ Cancer Stem Cells, the epithelial one (EpCAM+) and the mesenchymal and metastatic one (CD133+CXCR4+ EpCAM- Metastasis initiating cells MICs). **(B)** FACS analysis of N=13 NSCLC primary tumors for the expression of CD73 within bulk tumor and different CSC subsets. **(C)** Comparison of CD73 expression by FACS analysis in n=4 primary tumors and synchronous lymph node metastases.

PD-L1 was significantly more expressed in CD133+ CSC subset (median value= 20%; min 2.5%, max 98%) compared to total population (median= 9.5%, min 0.5%, max 96%) (**Figure 1A**). Among CSC subsets, we could detect the population of mesenchymal CD133+EpCAM-CXCR4+ metastasis initiating cells (MICs) in 17 cases of primary tumors. Notably, we verified that it was the highest expressor of PD-L1 (median value= 31.8%; min 6%, max 100%). Conversely, CD133+ CSCs positive for the epithelial marker EpCAM showed lower expression of PD-L1 (median value = 16.6%; min 4%, max 98%) (**Figure 1A**).

We also observed a down-regulation of HLA class I, antigen presenting molecule, in CD133+ CSC compared to tumor bulk population, both in NSCLC primary tumors (n=6) and NSCLC cell lines (n=4) (0,7 fold decrease compared to bulk cells) (**Figures S1A, B**), confirming the ability of CSC to escape immune cells recognition. In 13 primary NSCLC samples, we also assessed CD73 expression within bulk population and CSC subsets. CD73 expression was significantly increased within the subset of CD133+CXCR4+ MICs (median value= 80%; min 14%, max 100%) compared to CD133+ CSCs (median= 44%; min 2%, max 95%) and bulk tumor (median= 46%; min 2.5%, max 65%) (**Figure 1B**).

Finally, in 4 cases, we were able to analyse primary tumors and corresponding synchronous lymph node metastases. The subset of metastatic and immunosuppressive CD133+CXCR4+CD73+ MICs was 2.6 fold-enriched in metastasis compared to primary tumors (**Figure 1C**).

Overall, this immunophenotypic characterization of primary NSCLC indicates that CSCs and in particular the fraction of MICs displays high levels of molecules involved in immune suppression.

### Lung Cancer Spheroids Are Enriched in MICs and Express Immunosuppressive Molecules

To study *in vitro* the immunosuppressive properties of MICs, we exploited a well-known method adopted to enrich for CSC population through the generation of cancer spheroids grown in selective medium, containing EGF and bFGF (45). We generated spheroids from 4 NSCLC cell lines: A549 and H3122 (adenocarcinoma), H1299 (large cell carcinoma), and SW900 (squamous cell carcinoma) (**Figure S2**). They were characterized for CD133+CXCR4+ phenotype, PD-L1, HLA class I, and for CD73, CD38, and CD39 expression, involved in the production of immunosuppressive adenosine (29).

**Figure 2:**
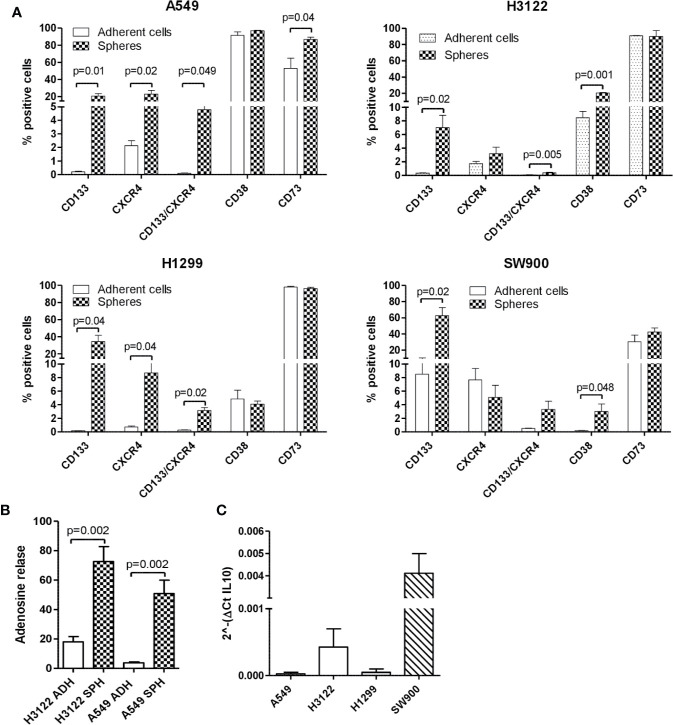
Lung spheroids are enriched in MICs and expressed high level of immunosuppressive markers then adherent cells. **(A)** FACS analysis of adherent NSCLC cell lines (A549, H3122, H1299, SW900) and corresponding spheroids for expression of CD133, CXCR4, CD38, and CD73 markers. Data are the mean value ± SD of n=4 analyses for each cell line. **(B)** AMP substrate was added to culture medium and generation of adenosine was quantified by HPLC in the medium of spheroids and adherent cells (A549 and H3122 cell lines). Data are the mean value ± SD of n=2 analyses for each cell line. **(C)** IL-10 gene expression evaluated by Real-Time PCR in lung spheroids cultures. Bar are the mean value ± SD of 2 ^- (CT IL-10-CT B2m)^.

Overall, compared to their parental adherent cell lines, spheroids were highly enriched in CD133+CXCR4+ MICs subset (30 fold-change), generally associated with an increase of either CD73 or CD38 markers (respectively 1.2 and 3 fold-change), both involved in immune regulation and generation of adenosine (**Figure 2A**). The expression of CD39, the ectoenzyme that functions in tandem with CD73 in the canonical adenosinergic pathway, was undetectable both in adherent cells and spheroids, suggesting that in our *in vitro* condition CD38/CD73 non-canonical pathway is uniquely responsible for adenosine production.

Finally, no significant modulation of PD-L1 or HLA class I was observed in any spheroids cell lines compared to parental adherent one (data not shown). To address whether the increase in CD73/CD38 observed by flow cytometry analysis in CSC-enriched spheroids could be functionally associated with an increased production of ADO, we added AMP to adherent cells and sphere cultures and quantified adenosine production by HPLC. Results showed an increase of adenosine levels in medium from spheroids compared to adherent cells (**Figure 2B**). These data suggested a direct connection between high membrane expression of CD73/CD38 and production of adenosine.

We also investigated the modulation of IL-10, a cytokine known to trigger immunosuppressive effects by inducing T reg and pro-tumorigenic macrophages. Gene expression Real-Time analysis showed that spheroids expressed different levels of IL-10, whereas in all tested adherent cell lines IL-10 expression was undetectable (**Figure 2C**).

Overall, our results show that spheroids generated *in vitro* can be exploited to investigate the immunosuppressive phenotype of MICs.

### Inhibition of CXCR4 Pathway Prevents Tumor Dissemination and Reduces Expression of Immunosuppressive Molecules

To block migration of CD133+CXCR4+ MICs, we tested a novel peptide inhibitor of CXCR4, peptide R, an analogue of SDF-1 (44).

**Figure 3:**
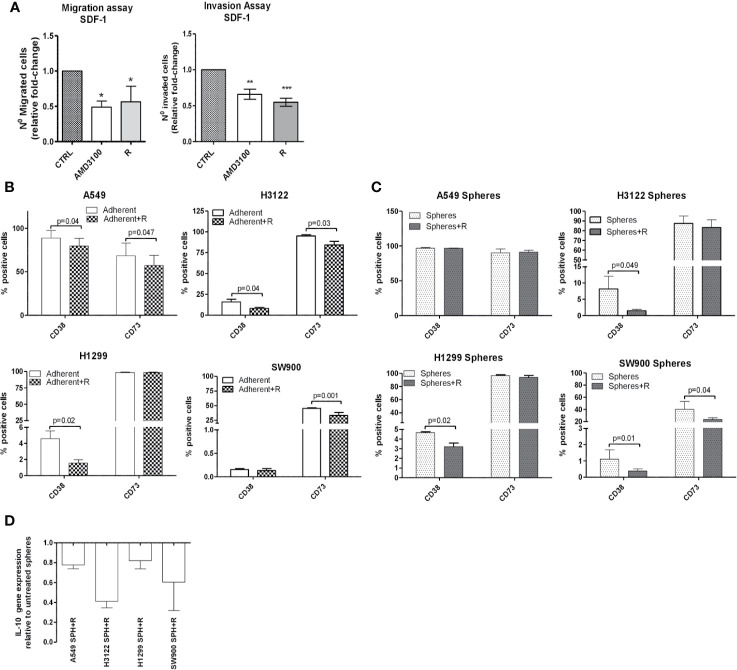
Inhibition of CXCR4 axis prevents MICs migration and decreases immunomodulatory marker expression. **(A)** Migration and invasion assay performed *in vitro* on A549, H3122, H1299 cell lines. Cells were treated with CXCR4 inhibitors: peptide R 1µM or AMD3100 10 µM and chemoattracted by SDF-1 50 ng/ml. Data represent the median fold change of number of migrated/invaded cells after treatment relative to untreated control. Duplicate experiments were performed for each cell line *p < 0.05. **p < 0.01. ***p < 0.001 **(B)** FACS analysis of adherent cells and **(C)** lung spheroids for the expression of CD38 and CD73 markers 2 h after treatment with peptide. Data are the mean value ± SD of n=3 analyses for each cell line. **(D)** Real-Time PCR quantification of IL-10 gene expression in spheroids after treatment with CXCR4 inhibitor compared to untreated cells.

Firstly, we assessed the ability of peptide R (1μM) to prevent both migration/invasion induced by SDF-1, similarly to AMD3100, a CXCR4 antagonist that has been clinically approved (**Figure 3A**). The experiments were performed in our panel of lung cancer cell lines.

We analyzed the phenotype of adherent cell lines after treatment with peptide R. Notably, we observed a reduced expression of markers, such as CD38 and CD73 (**Figure 3B**). We verified that the modulation of CD38 and CD73 expression induced by CXCR4 blockade was a rapid event, with the greatest effect observed 2 h post treatment and that rapidly reverted to basal expression (**Figure S1A**). We also confirmed the down-regulation of CD73 after CXCR4 inhibition by WB and Real-Time analyses (**Figures S3B, C**).

However, since in adherent cell lines only a small percentage of cells expressed CXCR4 (median value 1.2%; min 0.7%, max 4.8%), we speculated that in lung cancer spheroids, highly enriched for CXCR4+ cells (median value 7.5%; min 2%, max 31.6%), treatment with peptide R might result in a more marked effect. Indeed, we proved that short-term treatment of spheroids with CXCR4 inhibitor was able to significantly reduce the expression of CD38 and/or CD73 in all cell lines (except for A549), likely indicating an adenosine decrease, and average 50% decrease of immunosuppressive IL-10 cytokine expression in all cell lines (**Figures 3C, D**). These results suggest a link between CXCR4 pathway and induction of immunosuppressive phenotype in MICs.

### CXCR4 Axis Inhibition Partially Rescues T Cells Suppression Caused by MICs

To functionally prove the relevance of effects on immunosuppressive molecules modulation induced by CXCR4 inhibition, we tested the ability of CM collected from treated cell lines versus untreated controls, in both adherent and spheroids condition, to induce T cell suppression.

**Figure 4:**
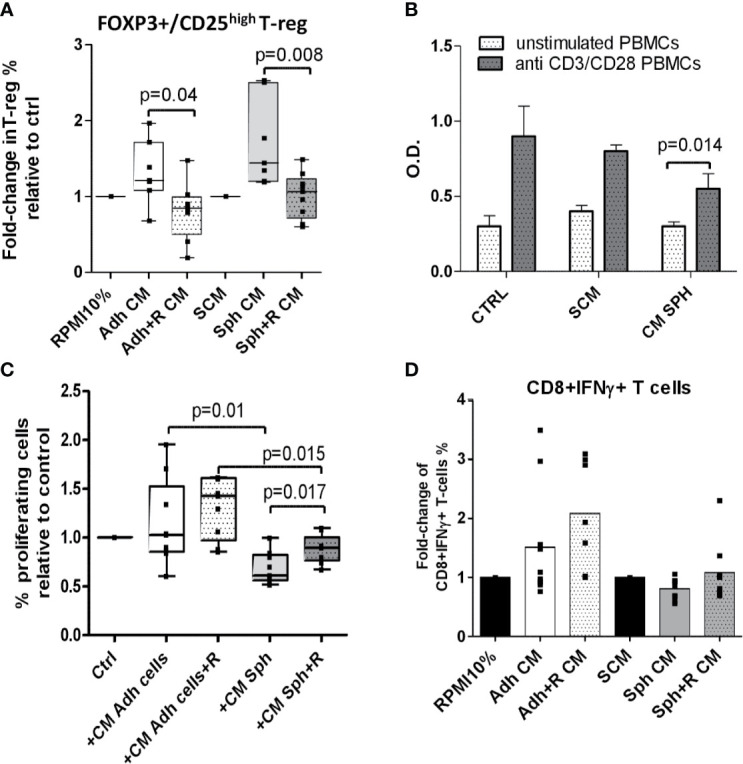
CM from spheroids induces T cells suppression that can be prevented by CXCR4 inhibition. **(A)** FACS analysis for Treg population within T lymphocytes, from N=8 healthy volunteers. T lymphocytes were stimulated with anti CD3/CD28 beads and incubated for 72 h with CM from adherent or spheroids cell lines, untreated or treated with peptide R. Data are the fold-change in % T reg population compared to proper control medium (RPMI 10% for adherent cells and Stem cells medium- SCM- for spheroids). Data are the mean value ± SD. N=2 independent experiments were performed for each tested NSCLC cell lines (A549/H3122/H1299/Sw900). **(B)** MTT assay measuring the proliferation of healthy volunteer T cells, unstimulated or stimulated with anti-CD3 and anti-CD28 antibodies, after exposure for 72 h to CM from A549 and H3122 spheroids or control RPMI or SCM medium for 72 h. Data are the mean value ± SD of N=4 independent experiment for each cell line. **(C)** CSFE assay measuring proliferation of healthy volunteers T cells, stimulated with anti-CD3 and anti-CD28 microbeads, after exposure for 72 h to CM from adherent or spheroids, treated or not with peptide R. Data are the fold-change in % of proliferating cells compared to proper control medium (RPMI 10% for adherent cells and SCM for spheroid). Data are the mean value ± SE of each NSCLC cell line (A549/H3122/H1299/SW900) tested in triplicate experiment. **(D)** FACS analysis for CD8+ T cytotoxic cells expressing IFNγ in N=8 healthy volunteers incubated for 72 h with CM from adherent or spheroids cell lines, untreated or treated with peptide R. Data are the fold-change in % CD8 T cytotoxic population compared to proper control medium (RPMI 10% for adherent cells and Stem cells medium- SCM- for spheroids). Data are the mean value ± SE of N=2 independent experiments were performed for each tested NSCLC cell lines.

Firstly, we assessed the effects of CM from adherent cells and spheroids in modulating T cells having regulatory function (T reg: CD4+Foxp3+CD25^high^). T cells from healthy volunteers were stimulated with anti CD3/CD28 micro beads and cultured in presence of CM from cancer cells lines. We showed that spheroids CM were able to increase the percentage of T reg compared to control, at higher extent than adherent cells (respectively 1.6 and 1.3 fold-increase). Notably, blockade of CXCR4 in both adherent and spheroid cells was sufficient to prevent the increase of T reg population induced by untreated counterpart (**Figure 4A**).

Next, since MIC-enriched spheroids were able to induce T reg phenotype, we assessed their potential to suppress T-cell activity. We demonstrated that lung spheroid CM were able to significantly suppress the proliferation of T cells, isolated from healthy donors PBMCs, after stimulation with anti CD3/CD28 antibodies (**Figure 4B**). When we compared the effect of spheroids and adherent cells, we observed that T cells from healthy donors proliferated significantly less in the presence of spheroids CM than adherent cells CM, and importantly, CM from spheroids treated with peptide R partially counteracted the suppressive effect on T cells (**Figure 4C**).

Finally, we verified that CM from spheroids were able to partially suppress (0.8 fold-change) the release of IFN-γ from CD8+ T cytotoxic cells (**Figure 4D**), derived from PBMCs of healthy volunteers, whereas CM from adherent cancer cell lines did not. CM from adherent and spheroid cancer cell lines treated with peptide R were able to relieve suppression of T cells and increase the subset of CD8+ T cells expressing IFN-γ compared to untreated cells (**Figure 4D**).

Overall, our data functionally prove that spheroids enriched in MICs possess an enhanced ability to suppress T-cell activity, concomitantly with the above reported increase in adenosine and IL-10 production. CXCR4 blockade is able to impair MIC immune suppression activity, preventing T reg generation and rescuing T cell activity.

### CXCR4 Inhibition Impairs CSC Ability to Promote TAM Polarization

Finally, we tested the ability of CM from lung cancer cell lines treated or not with peptide R to induce M0 macrophages polarization toward tumor-associated macrophages (TAM), known to possess immunosuppressive properties (46).

Macrophage cultures were derived from healthy volunteers. We evaluated by FACS the increased percentage of CD206+, CD163+, and CD14-CD206+ cell subsets and by Real-Time PCR an increased expression of IL-10, VEGF, and, conversely, a decreased expression of pro-inflammatory cytokines IL-12 and IL-6 as a read out of the induction of TAM phenotype after exposure to cancer cells CM, as reported by Benner et al. (47).

**Figure 5:**
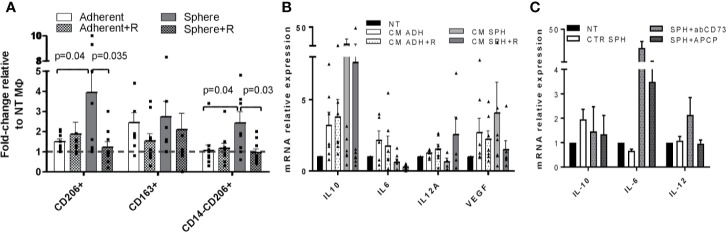
CM from spheroids induces TAM polarization that can be prevented by CXCR4 inhibition. **(A)** FACS analysis for CD206, CD163, and CD14 expression in macrophages derived from PBMCs of healthy volunteers treated with CM from adherent or spheroids, treated or not with peptide R. Data are the fold-change in % of positive cells compared to control macrophages cultured in proper control medium (RPMI 10% for adherent cells and SCM for spheroids). N=2 independent experiments were performed for each tested NSCLC cell lines. **(B)** Real-Time PCR quantification of IL-10, IL-6, IL-12, VEGF gene expression in macrophages derived from PBMCs of heavy smoker volunteers treated with CM from adherent or spheroids, treated or not with peptide R. Control macrophages cultured in proper control medium (RPMI 10% for adherent cells and SCM for spheroids) were used as calibrator. N=2 independent experiments were performed for each tested NSCLC cell lines. **(C)** Real-Time PCR quantification of IL-10, IL-6, IL-12 genes expression in macrophages derived from PBMCs of heavy smoker volunteers treated with CM from spheroids, untreated or treated with anti CD73 antibody or APCP. Control macrophages cultured in SCM were used as calibrator.

Despite the variability across macrophage cultures from different volunteers, we found that CM from spheroid cell lines enriched in MICs were more prone to induced TAM polarization compared to adherent cell lines, confirming the immunosuppressive behavior of MICs (**Figures 5A, B**). Indeed, CM from spheroids proficiently expanded the subset of CD206+/CD163+ and CD14-CD206+ macrophages **Figure 5A**) and induced the up-regulation of IL-10 and VEGF with a concomitant decrease of IL-12 and IL-6 (**Figure 5B**), a phenotype typically associated with TAM.

To exclude that different medium composition (RPMI 10% or SCM) could drive modulation of immune regulation induced by cancer cells, we treated macrophage cultures with both RPMI 10% or SCM media conditioned by adherent NSCLC cell lines. We verified that the effects of the two CMs in the induction of TAM phenotype were very similar, indicating that different medium composition does not modify the intrinsic ability of cancer cells to induce TAM polarization (**Figure S4A**).

Finally, we assessed whether the observed increased immunosuppressive effects of spheroids reflect specific properties of selected cancer cells or can be related to the different *in vitro* culture conditions (adherent vs suspension). We exploited the immortalized but not tumorigenic human bronchial epithelial cell line (HBEC3-KT) that is not expected to induce an immunosuppressive effect on PBMCs. HBEC cells were grown in adhesion and in suspension as spheroids and SCM conditioned medium was collected by both cultures. Macrophages treated with SCM-CMs from both adherent and spheroids HBEC failed to show TAM polarization, as assessed by FACS and Real-Time PCR analyses (**Figure S4B**).

Overall, these data confirm that differences observed between CM from adherent and spheroids NSCLC cell lines are not due to medium composition or different culture conditions, but instead related to the intrinsic properties of spheres enriched in MIC population, with higher potential to induce immunosuppressive effects.

The treatment of lung spheroid cultures with peptide R was able to partially prevent TAM polarization, significantly reducing CD206/CD163 surface expression and IL-10, VEGF gene expression while increasing IL-12 production compared to untreated control (**Figures 5A, B**).

To prove the role of adenosine as a key mediator of immunosuppressive properties of MICs, we treated spheroids with the Adenosine 5’-(α,β-methylene)diphosphate (APCP) and with a neutralizing antibody against CD73, both able to impair adenosine production (29, 32). We observed that collected media from Ab-treated cells were able to revert TAM phenotype induced by lung cancer spheroids as indicated by a decrease of IL-10 and an increase of IL-6 and IL-12 (the latter was observed only after moAb treatment) (**Figure 5C**).

Overall, our data suggest that MICs-enriched spheroids not only directly act on T cell regulation but also induce polarization of TAM, which can exacerbate an immune suppressive environment.

## Discussion

Cancer stem cells are composed of heterogeneous populations, each with a specific function (48, 49). The subset of CSCs deputed to metastasis initiation possesses features allowing primary tumor escape, survival in circulation, and distant organs seeding (50, 51). Immune escape mechanisms adopted by MICs are supposed to be essential to complete all the steps leading to metastasis generation (52, 53).

Some evidence has reported that CSCs are characterized by specific immunological properties, which protect them against chemotherapeutic drugs but also increase their resistance toward apoptosis-inducing immune effectors, like T or NK cells (54). Several mechanisms can be exploited by CSCs to escape immune surveillance, such as down-regulation of MHC class I and II molecules, inefficient antigen presentation, and release of immunosuppressive factors (52). These strategies would help CSCs to survive, sustain tumor progression, and metastasize (53).

Moreover, it has been reported that there is a correlation between immunosuppressive environment and activation of epithelial to mesenchymal transition (EMT) program, endowing primary tumor cells with disseminating and stemness properties (52, 55). Dongre et al. showed that mesenchymal traits of tumor cells are associated with high levels of PD-L1 expression, content of T reg cells, and M2-like macrophages, proving that EMT activation in tumor cells promotes the recruitment of immunosuppressive cells and immune surveillance escape (56). In NSCLC the activation of EMT by the up-regulation of ZEB1 transcriptional factors causes the up-regulation of PD-L1 by tumor cells, leading to CD8+ T cells immune suppression and increased metastasis (57).

All together, these evidences suggest that a deeper understating of the immune profile of CSCs, and in particular of the mesenchymal subset deputed to metastasis initiation, can pave the way for specific anti-CSC immunotherapy, necessary to achieve a complete eradication of tumors and control of metastatic diseases.

In NSCLC, we previously showed that the population of CD133+/CXCR4+ MICs is endowed with stemness and EMT features, enhanced resistance to cisplatin, and superior ability to seed distant organs and initiate metastasis (11, 13). However, the immunological characterization of this subset has never been reported.

Here, we show that NSCLC MICs express the highest levels of both PD-L1 and CD73, compared to bulk tumor cells and epithelial CSC subset, suggestive for increased potential to suppress T cell activity.

An increased expression of PD-L1 has also been reported in CSCs of other tumor types. In head and neck carcinomas the subset of CD44+ CSCs expressing high level of PD-L1 can selectively evade host immune responses. The use of an immune check point inhibitor against PD-1 partially restored the immunogenicity of CD44+CSCs, providing the rationale for an anti CSC-immunotherapy (58).

In triple negative breast cancer, ALDH/CD44+ CSCs exhibited increased levels of PD-L1 versus non-CSC tumor cells. ALDH/CD44+/PD-L1+ CSCs were found in close contact with PD-1+ T cells both in murine and human tumor samples, suggesting a direct effect of CSCs in immune control (59).

In our study, we report that NSCLC CSCs co-expressing CXCR4 and CD73 are enriched in lymph node metastasis compared to primary tumors, indicating that the cells able to initiate metastasis may have an enhanced immunosuppressive activity.

This result confirms previously published literature reporting increased CD73 levels in metastatic tumors (31, 60). Moreover, studies deriving from different solid tumors reported CD73 expression as a poor prognostic factor (37, 61), suggesting that CD73-adenosine pathway plays a fundamental role in tumor dissemination, likely promoting immune suppression.

To investigate *in vitro* the immunosuppressive phenotype of CD133+/CXCR4+ CSCs overcoming the limitation of the paucity of CSCs in established adherent NSCLC cell lines, we adopted the sphere forming assay, a method commonly recognized to enrich for CSC subset (45). Spheroid cultures generated from NSCLC cell lines recapitulate the immunosuppressive phenotype of CD133+CXCR4+ MICs subset, also expressing higher levels of CD73 and CD38 as compared to adherent cells.

Adenosinergic signaling is a physiopathological regulator of tissue homeostasis, particularly upon injury and stress. Indeed, adenosine rapidly increases in response to stress, hypoxia, or tissue injury inducing repair processes (62). High levels of extracellular adenosine, generated by canonical CD39/CD73 or non-canonical CD38/CD73 adenosinergic pathways in tumor microenvironment (28, 29), can promote tumor progression by directly stimulating tumor proliferation, migration, invasion, and metastatic dissemination and by favoring immune escape of tumor cells (33). From a functional point of view, CD133+CXCR4+ MICs subset showed an increased release of immunosuppressive adenosine, due to the activation of CD38/CD73 pathway, and indeed CD38 and CD73 resulted highly expressed, while CD39 expression was undetectable Further, we also detected the increase release of IL-10, known to trigger immunosuppressive effects by inducing T reg cells and pro-tumorigenic immunosuppressive polarization of macrophages (63).

When we functionally tested *in vitro* immune regulatory properties of lung spheroids and corresponding adherent cells, we demonstrated that MICs-enriched spheroids possess an increased ability to induce T reg cells and consequently to suppress T cell proliferation as well as to reduce cytotoxic ability of CD8+ T cells.

Similarly, it was demonstrated that CSCs from glioblastoma inhibited T cell proliferation of healthy donors and showed lower immunogenicity and higher suppressive activity compared to corresponding adherent cell lines (64).

We also assessed the effect of spheroids to induce polarization of macrophages toward TAM phenotype that are very well known to promote immune suppression, tumor cell invasion, and metastasis (46, 65).

Conditioned media from cancer cells can be exploited to induce TAM polarization (47). In particular, TAM phenotype is associated with a high expression of immunosuppressive IL-10 and pro-angiogenic VEGF and low levels of inflammatory cytokines (IL-6 and 12). Besides, there is generally an increase of CD206/CD163 markers and reduced CD14 surface expression (47). All of these features were detected in cultures of macrophages derived from PBMCs of volunteers exposed to spheroids CM, thus *bona fede* providing support to the ability of MICs to induce TAM polarization that can exacerbate immunosuppressive environments.

It has been previously reported that one of the pathways stimulated by adenosine is the up-regulation of CXCR4 in cancer cells, increasing their ability to migrate and proliferate in response to CXCL12 (17). CXCR4 expression is an important factor for maintenance of stemness and endowment of metastatic potential of NSCLC CSCs (66). Thus, targeting CXCR4 could be useful both to block CSCs and to decrease tumor microenvironment immune suppression.

Moreover, CXCR4 is highly expressed also by the subset of immunosuppressive Treg cells. CXCR4 and its inhibition have been demonstrated in different tumor types to efficiently revert Treg suppression of T effectors proliferation, improving anticancer immune responses (23, 67).

CXCR4/CXCL12 axis inhibition has been demonstrated to revert tolerogenic polarization of tumor microenvironment (42) and to restore sensitivity to CTLA-4 and PD-1 ICIs (23, 43), overall representing a novel and effective way to counteract ICIs resistance. In the present study, we tested a novel peptide inhibitor of CXCR4, peptide R, analogue of CXCL12 (44), to target CD133+CXCR4+ MICs. We show that the treatment of NSCLC spheroids with Peptide R, besides preventing tumor cell dissemination, decreases expression of immunosuppressive molecules, such as CD73, CD38, and IL-10.

Furthermore, the functional blockade of CXCR4 in tumor cells is sufficient to prevent the immunosuppressive ability of MICs by restoring T cell proliferation and IFN_γ_ expression, as well as partially preventing TAM polarization.

Our study has some limitations, mainly related to the small effects observed among treatment groups and the lack of *in vivo* validation of the findings. Indeed, treatment of PBMC with CM in some experiments resulted in biological effects that did *not* reach *statistical significance* mainly due to the great variability among PBMC from different healthy volunteers and to the use of several NSCLC cell lines. Despite the expected variability, we decided to test different NSCLC cell lines to take into consideration the heterogeneity of NSCLC histological subtypes and to avoid the potential bias of single cell line-dependent effects.

*In vivo* validation of our observation could definitely strengthen our conclusions. However, the *in vivo* investigation of the immunosuppressive ability of human tumor cells is hampered by the necessity to use immunocompromised mice, lacking adaptive immunity, to grow xenograft tumors. The establishment of a more sophisticated humanized murine model reconstituted with human immune cells might provide in the near future further validation of our *in vitro* evidence.

Finally, the validation of the potential of CXCR4 blockade to counteract MICs immune escape may be challenging *in vivo.* Since CXCR4 is wildly expressed both by tumor and stroma/immune cells, the systemic delivery of CXCR4 inhibitors *in vivo* could affect these different cell subsets, impairing the possibility to finely dissect the players involved in the generation of the immunosuppressive microenvironment and the impact of CXCR4 inhibition on this tumor-stromal crosstalk.

Despite these limitations, taken together our data suggest the high ability of MICs to escape immune control and corroborate the link between CXCR4 pathway and the induction of immunosuppressive phenotype in CSCs. Consequently, they point at CXCR4 inhibitors as potential innovative agents to implement efficacy of immunotherapy, by concurring in reverting immune suppression and preventing metastatic dissemination.

## Data Availability Statement

All datasets presented in this study are included in the article/**Supplementary Material**.

## Ethics Statement

The studies involving human participants were reviewed and approved by IRB of Fondazione IRCCS Istituto Nazionale dei Tumori. The patients/participants provided their written informed consent to participate in this study.

## Author Contributions

GB and IR conceived the study. OF, DB, FG, MC, CB, AH, IR, and GB performed the experiments and analyzed the data. LR, IR, and GB supervised data acquisition and analysis. UP provided clinical samples. SS provided the CXCR4 inhibitor. GB, IR, and OF wrote the manuscript. MC, AH, FM, RF, SS, GS, and LR reviewed and critically assessed the manuscript. All authors contributed to the article and approved the submitted version.

## Funding

This work is supported by Associazione Italiana per la Ricerca sul Cancro (AIRC) (grant number IG21431 to LR and IG18812 to GS); Roche Italia (Roche per la Ricerca 2017 GB); CRT Foundation (CRT ordinary grant 2018 to IR).

## Conflict of Interest

The authors declare that the research was conducted in the absence of any commercial or financial relationships that could be construed as a potential conflict of interest.
