# Supplementary material for: CXCR4 Inhibition Counteracts Immunosuppressive Properties of Metastatic NSCLC Stem Cells
Source: Front Immunol. 2020 Oct 2;11:02168. doi: 10.3389/fimmu.2020.02168 (PMC7566588; doi:10.3389/fimmu.2020.02168)
Supplement: Supplementary file 1 [file DataSheet_1.docx]

Supplementary Material

## SUPPLEMENTARY FIGURES

**Supplementary Figure 1. CSCs down-modulate HLA class I expression. (A)** FACS analysis of n=6 NSCLC primary tumor for HLA ABC expression within bulk tumor and CD133+ CSC subset. (B) FACS analysis for HLA ABC, as in A, performed on NSCLC cell lines (A549, H3122, SW900, H1299). N=4 replicates independent analysis for each cell line.

**Supplementary Figure 2. Representative images of adherent NSCLC cell lines (on the left) and corresponding spheroids, grown in suspension (on the right)**

**Supplementary Figure 3. CXCR4 inhibitor rapidly induces the down-modulation of CD73 and CD38. (A)** FACS analysis for CD38 and CD73 expression in A549 and H1299 cell line treated with peptide R for 1, 2, 5 or 24h. Data are the mean fold change of % positive cells relative to untreated control, n=2 for each time points. **(B)** Representative WB analysis for CD73 of H3222 adherent cell line, in untreated control and after 1 ,2, 5 and 24 h of incubation with peptide R, CXCR4 inhibitor. **(C)** Real-Time PCR quantification of CD73 gene expression in adherent cells lines after 2h treatment with CXCR4 inhibition compared to untreated cells.

**Supplementary Figure 4. Different medium composition or culturing conditions do not impact the cell ability to induceTAM polarization. (A) On the left:** FACS analysis for CD206, CD163 and CD14 expression in macrophages derived from PBMCs of healthy volunteers treated with RMPI and SCM conditioned by adherent A549, H3122, H1299, SW900 cells. Data are the fold-change in % of positive cells compared to control macrophages cultured in proper control medium. N= 4 experiments, one for each cell line. **On the right**: the same treated macrophages were analyzed by Real-Time PCR to quantify IL-10, IL-6, IL-12, VEGF gene expression. Control macrophages cultured in proper control medium (RPMI 10% for adherent cells and SCM for spheroids) were used as calibrator. **(B) On the left:** FACS analysis for CD206, CD163 and CD14 expression in macrophages derived from PBMCs of healthy volunteers treated with SCM conditioned by adherent and spheroids HBEC cells. Data are the fold-change in % of positive cells compared to control macrophages cultured in non-conditioned SCM medium. N= 2 experiments. **On the right:** the same treated macrophages were analyzed by Real-Time PCR to quantify IL-10, IL-6, IL-12, VEGF gene expression. Control macrophages cultured in SCM control medium were used as calibrator.
